# T Cells Are Dominant Population in Human Abdominal Aortic Aneurysms and Their Infiltration in the Perivascular Tissue Correlates With Disease Severity

**DOI:** 10.3389/fimmu.2019.01979

**Published:** 2019-09-04

**Authors:** Agnieszka Sagan, Tomasz P. Mikolajczyk, Wojciech Mrowiecki, Neil MacRitchie, Kevin Daly, Alan Meldrum, Serena Migliarino, Christian Delles, Karol Urbanski, Grzegorz Filip, Boguslaw Kapelak, Pasquale Maffia, Rhian Touyz, Tomasz J. Guzik

**Affiliations:** ^1^BHF Cardiovascular Research Centre, Institute of Cardiovascular and Medical Sciences, University of Glasgow, Glasgow, United Kingdom; ^2^Department of Internal and Agricultural Medicine, Jagiellonian University Medical College, Kraków, Poland; ^3^Centre for Immunobiology, Institute of Infection, Immunity and Inflammation, University of Glasgow, Glasgow, United Kingdom; ^4^Department of Vascular Surgery, CUMRiK, University Hospital, Kraków, Poland; ^5^Department of Vascular Surgery, Queen Elizabeth University Hospital, Glasgow, United Kingdom; ^6^Department of Cardiovascular Surgery and Transplantology, John Paul II Hospital, Kraków, Poland; ^7^Institute of Cardiology, Jagiellonian University Medical College, Kraków, Poland; ^8^Department of Pharmacy, University of Naples Federico II, Naples, Italy

**Keywords:** abdominal aortic aneurysm, T cell, perivascular adipose tissue, inflammation, macrophages

## Abstract

Abdominal Aortic Aneurysm (AAA) is a major cause of cardiovascular mortality. Adverse changes in vascular phenotype act in concert with chronic inflammation to promote AAA progression. Perivascular adipose tissue (PVAT) helps maintain vascular homeostasis but when inflamed and dysfunctional, can also promote vascular pathology. Previous studies suggested that PVAT may be an important site of vascular inflammation in AAA; however, a detailed assessment of leukocyte populations in human AAA, their anatomic location in the vessel wall and correlation to AAA size remain undefined. Accordingly, we performed in depth immunophenotyping of cells infiltrating the pathologically altered perivascular tissue (PVT) and vessel wall in AAA samples at the site of maximal dilatation (*n* = 51 patients). Flow cytometry revealed that T cells, rather than macrophages, are the major leukocyte subset in AAA and that their greatest accumulations occur in PVT. Both CD4^+^ and CD8^+^ T cell populations are highly activated in both compartments, with CD4^+^ T cells displaying the highest activation status within the AAA wall. Finally, we observed a positive relationship between T cell infiltration in PVT and AAA wall. Interestingly, only PVT T cell infiltration was strongly related to tertiles of AAA size. In summary, this study highlights an important role for PVT as a reservoir of T lymphocytes and potentially as a key site in modulating the underlying inflammation in AAA.

## Introduction

Abdominal aortic aneurysm (AAA) is defined as a pathological dilatation of the aorta, to more than 1.5 times the normal diameter. AAAs are one of the most important causes of cardiovascular morbidity and mortality and occurs in up to 9% of men after 65 years of age (1). AAA shares many of the same risk factors as atherosclerosis including advanced age, smoking, hypertension, and hypercholesterolemia (2). Recently, it has also been shown that elevated BMI also increased the likelihood of AAA diagnosis (3). The mechanisms of AAA, defined primarily in animal model studies, are complex, involving smooth muscle cell apoptosis, oxidative stress (4), and inflammation (5). Clinically, patients with AAA have elevated circulating pro-inflammatory cytokines (6–8) and immunohistochemical studies of AAA reveal the presence of inflammatory cells such as macrophages, T cells, B cells, dendritic cells, natural killer cells, neutrophils, and mast cells (9–14).

Amongst inflammatory cells, macrophages are an important subpopulation with their role in the pathogenesis of AAA well-described in both mice and humans (15–21). Furthermore, T and B cell numbers are increased in cryosections of aneurysmal tissues (13, 22) with lymphocyte density negatively correlating with collagen and elastin content indicating a contribution of adaptive immune cells to AAA instability (14). Both Th1 (23–25) and Th2 (26) CD4^+^ T cells as well as CD8^+^ T cells have been implicated in promoting AAA formation (23, 27, 28). Despite the evidence of the presence of these cells in aneurysmatic vascular wall (23, 26–30), less is known regarding their respective number, activation status and spatial distribution within the vessel wall. While circulating pro-inflammatory CD4^+^ T cells are increased in patients with moderate sized AAA (24), it is uncertain if there is a relationship between aortic wall T cell content and AAA size.

While most studies focus on alterations in the vascular wall of AAA, recent interest in the role of perivascular adipose tissue (PVAT) inflammation and its clinical significance (31–33) raise an important question on the role of perivascular tissue (PVT) in AAA. PVT regulates vascular function; however, imbalances between the production and release of protective factors and pro-inflammatory molecules in PVT may result in vascular pathology (34, 35). In various cardiovascular and metabolic diseases such as atherosclerosis, hypertension, diabetes, or obesity, dysfunctional PVT plays a critical role, characterized by oxidative stress and inflammation (32, 36, 37). Furthermore, gene expression analysis suggested increased infiltration of immune cells into PVT surrounding AAA (38). Therefore, it is essential to characterize immune cell subpopulations infiltrating PVT surrounding AAAs and address their potential functional implications for aneurysm progression and size.

Here, we utilized a pool of clinical AAA samples to perform a quantitative assessment of leukocyte subsets, of which T cells are most abundant. We also focused on their presence in PVT, aiming to understand their links to AAA wall infiltration as well as the relationship to AAA size.

## Methods

### Human Samples

Segments of AAA were obtained during AAA repair surgery at the site of maximal dilatation from 51 patients. Clinical data including major risk factors for atherosclerosis and AAA were recorded at the time of surgery (Table 1). Hypercholesterolemia was defined as a plasma TC > 4.8 mM or the use of cholesterol lowering medication. Patients were considered hypertensive if BP was >140/90 mmHg or if patients were currently taking BP lowering medication. Diabetes was diagnosed based on a fasting glucose >5.5 mM or current treatment with insulin or oral hypoglycaemic agent (39).

**Table 1:** Patient clinical parameters including risk factors and current treatment regimens recorded at the time of surgery.

***N***	**51**
Age (years, mean ± SD)	67.9 ± 8
Sex (M:F)	42:9
Present thrombus (*n*, %)	44 (86%)
Aneurysms diameter (mm, mean ± SD)	62 ± 14
**RISK FACTORS**	
Smoking (*n*, %)	36 (70%)
Hypertension (*n*, %)	40 (78%)
Systolic BP (mmHg, mean ± SD)	129.3 ± 15
Diastolic BP (mmHg, mean ± SD)	79.1 ± 7.9
Hypercholesterolemia (*n*, %)	47 (92%)
Total Cholesterol (mmol/L, mean ± SD)	4.9 ± 1.27
Overweight/Obesity (*n*, %)	31 (61%)
BMI (kg/m^2^, mean ± SD)	26.25 ± 3.7
Diabetes (T2) (*n*, %)	7 (14%)
**MAIN MEDICATIONS**	
Diuretics (*n*, %)	20 (39%)
ACE inhibitors/ARB (*n*, %)	32 (63%)
ASA (*n*, %)	38 (74%)
Other antithrombotic (*n*, %)	7 (14%)
β blockers (*n*, %)	26 (51%)
Calcium antagonist (*n*, %)	8 (16%)
HMG CoA Inhibitors (*n*, %)	43 (89%)

*ASA, acetylsalicylic acid; BMI, body mass index; ACE, Angiotensin Converting Enzyme; HMG CoA Inhibitor, hydroxy-3-methyl-glutaryl-CoA reductase inhibitor*.

AAA size was determined in pre-operative CT and verified intraoperatively. Immediately after harvesting, samples were placed in ice-cold (4°C) phosphate buffered saline (PBS, Gibco, Invitrogen, Carlsbad, CA, USA) and transported to the laboratory. Written informed consent was obtained from all patients. Collection of tissue was approved by the local Research Ethics Committee of the Jagiellonian University, Kraków, Poland (Approval No. KBET/78/B/2012) and the West of Scotland Research Ethics Service Committee for the Biorepository at the Queen Elizabeth Hospital, Glasgow, United Kingdom (Approval No. 10/S/0704/60). Importantly, due to amount of tissue available and cell numbers in CD45^+^ gate, not all measurements were possible in all subjects and *n* numbers are provided in individual figure legends.

### Flow Cytometry Analysis of Cells in Tissues

In the laboratory, fragment of aneurysm was divided into two parts: wall (containing mostly intima-media) and PVT (containing PVAT and contiguous remodeled adventitia). Samples, were subsequently mechanically disrupted and digested with a cocktail of enzymes containing 125 U/ml collagenase type XI, 60 U/ml hyaluronidase type IVS and 450 U/ml collagenase type I (all from Sigma-Aldrich, Irvine, UK) in PBS with calcium- magnesium containing 20 mM Hepes at 37°C for 20 min with gentle agitation to isolate residual cells infiltrating tissues. The resulting cell suspension was then passed through a 70 μm strainer (BD Pharmingen, San Diego, CA, USA). Cells were incubated with fluorescently labeled antibodies for 20 min at 4°C (for details see Supplemental Table 1). Fluorescence Minus One (FMO) was used as negative control. After washing, cells were re-suspended in PBS with 1% fetal bovine serum (FBS) (Gibco, ThermoFisher Scientific, UK) and data acquired on a FACSCanto II cytometer (BD Bioscience, UK) and analyzed using FACSDiva™ and FlowJo software (Tree Star Inc, Olten, Switzerland).

### Immunofluorescence Staining

Immunofluorescence was performed on frozen 7 μm OCT- embedded aneurysmal tissue sections. For T cell visualization, rabbit polyclonal anti-human CD3 (ab5690; Abcam, Cambridge, UK) was employed and for macrophages, mouse monoclonal anti-human CD68 (ab955; Abcam, Cambridge, UK) was used. Appropriate secondary antibodies were employed (Donkey anti-rabbit IgG-Alexa Fluor 594 and Donkey anti-mouse IgG—Alexa Fluor 647, ThermoFisher Scientific). Sections treated with secondary antibodies alone did not show specific staining. Staining was visualized on a Zeiss Cell Observer SD confocal microscope (Zeiss, Oberkochen, Germany).

### Statistical Analysis

Patient clinical parameters are expressed as Mean ± SD as detailed in Table 1. Other data are expressed as Mean ± SEM except on dot plot graphs where data is expressed as Median (Q1;Q3). To test normality of distribution, Kolmogorov-Smirnov test was employed. Comparison between related samples were made using Wilcoxon matched pairs test, one-way ANOVA or *t*-test and between independent samples using Mann-Whitney test. Correlation between cells was assessed by Spearman's rank correlation analysis.

## Results

### Local Inflammation in Aneurysm Is Prone to Perivascular Tissue

**Figure 1:**
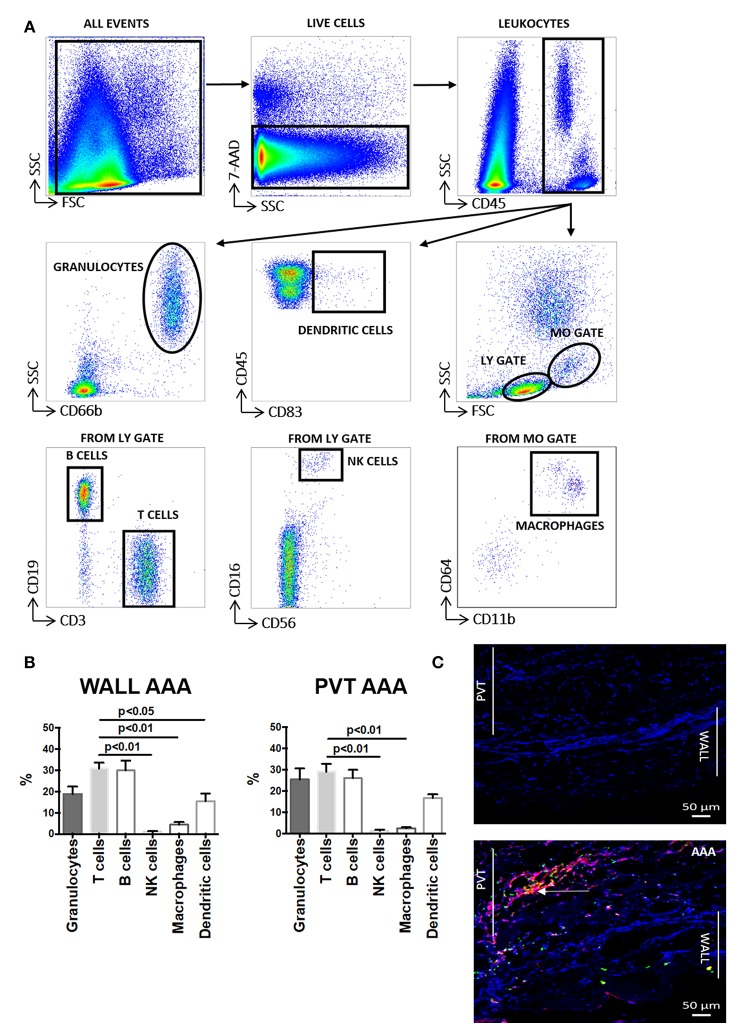
Aortic abdominal aneurysm (AAA) leukocyte infiltration: comparison and relationship between aneurysmal wall and PVT. **(A)** Gating strategy depicting identification of total leukocytes (CD45^+^) and leukocyte sub-populations: granulocytes, dendritic cells, B cells, T cells, NK cells and macrophages in AAA (aortic abdominal aneurysm). Gates were applied based on fluorescence minus one (FMO) analysis. **(B)** Distribution of the main leukocytes: T cells (CD3^+^), B cells (CD19^+^), NK cells (CD16^+^CD56^+^), macrophages (CD11b^+^CD64^+^), dendritic cells (CD83^+^), granulocytes (CD66b^+^) in wall and PVT of AAA tissue (*n* = 8–11), T cell percentages compared to other leukocyte subpopulations, *t*-test for related samples, *p* values presented on graphs only for statistically significant comparisons. **(C)** Bottom panel: example of immunofluorescence staining of T cells (CD3^+^) shown in red and macrophages (CD68^+^) shown in green in PVT and wall of an AAA. Nuclear staining (DAPI) is shown in blue. Example of T cell and macrophage co-localization shown by yellow/orange staining; white arrow. Representative of *n* = 5; Top panel: negative control consisting of secondary antibody staining only with DAPI. Scale bar = 50 μM.

**Figure 2:**
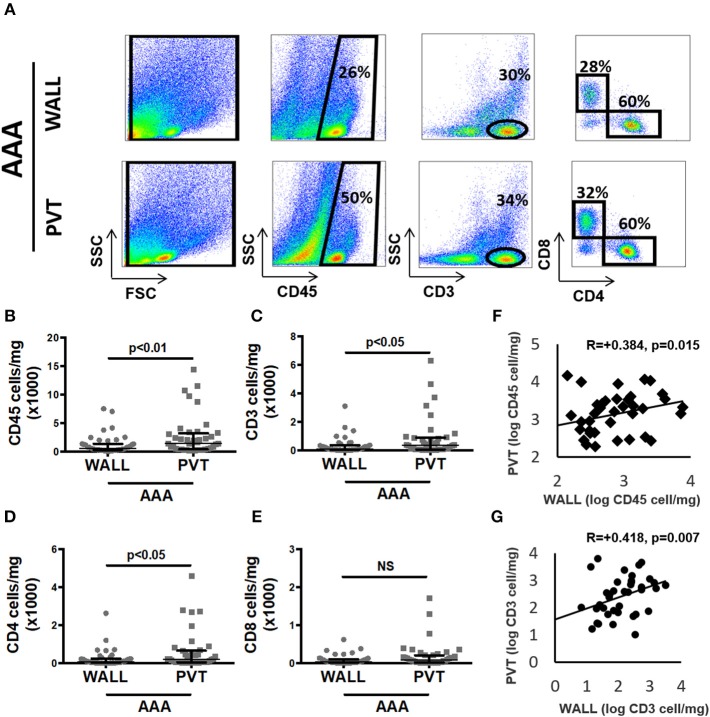
Aortic abdominal aneurysm (AAA) leukocyte infiltrate: comparison and relationship between aneurysmal wall and PVT. **(A)** Example of flow cytometric identification of leukocytes (CD45^+^), total T cells (CD3^+^) and CD4^+^, CD8^+^ T cell subpopulations in aneurysmal wall and PVT. **(B)** Leukocyte number per mg of aneurysmal wall vs. PVT, *n* = 40, **p < 0.01 (*Wilcoxon matched paired*). **(C)** T cells number per mg of aneurysmal wall vs. PVT, *n* = 39, *p < 0.05 (*Wilcoxon matched paired*). **(F)** Spearman rho correlation between number of leukocytes in aneurysmal wall and PVT; *R* = 0.384, *n* = 40, *p* = 0.015. **(G)** Spearman rho correlation between number of T cells in aneurysmal wall and PVT; *R* = 0.418, *n* = 39, *p* = 0.007. **(D)** CD4^+^ T cell number per mg of wall and PVT of AAA tissue, *n* = 39, *p* < 0.05 (*Wilcoxon matched paired*). **(E)** CD8^+^ T cell number per mg of wall and PVT in AAA, *n* = 39, NS (*Wilcoxon matched paired*).

We performed flow cytometry on cell suspensions to characterize leukocyte content and its subsets. The gating strategy used in these studies is presented in Figure 1A. We observed that following tissue digestion, absolute cell counts revealed a greater cellularity to the PVT layer in comparison with AAA wall (Supplemental Figure 1). Interestingly, analysis of the percentage distribution of leukocyte subpopulations in AAA shows that the major subpopulation are T cells for both aneurysmal PVT (29 ± 3%) and wall (31 ± 3%; Figure 1B) while other leukocyte subsets were less abundant, with a surprisingly low presence of macrophages in both AAA wall and PVT (Figure 1B). Immunofluorescence staining of AAA revealed an increased presence of T cells within PVT compared with AAA wall (Figure 1C). Interestingly, T cells and macrophages could also on occasion be found co-localizing within the PVT (Figure 1C). Quantifiable results obtained by flow cytometry revealed that the majority of leukocytes localized within aneurysmal PVT. Median (Q1;Q3) values for wall vs. PVT were, respectively, 578 (293;1353) vs. 1,428 (434;3137) cells/mg tissue (Figures 2A,B).

### T Cell Infiltration in AAA Perivascular Tissue

As T cells were the most abundant leukocyte subpopulation, we investigated them in more detail by showing that the T cell content in aneurysmal PVT was significantly higher in comparison to aneurysmal wall. Median (Q1;Q3) values were 109 (38;351) vs. 346 (69;862) cells/mg for wall and PVT, respectively (Figures 2A,C). In spite of these differences, we found a positive correlation between total leukocyte content in PVT and AAA wall *R* = 0.38, *p* = 0.015 (Figure 2F). A similar correlation was observed for T cell content *R* = 0.42, *p* = 0.007 (Figure 2G).

With regards to T cell subtypes, CD4^+^ T cells were significantly increased in PVT: 187 (41;580) cells/mg compared with wall 81 (20;226) cells/mg (Figure 2D). While a trend toward increased CD8^+^ T cells was observed in aneurysmal PVT, this did not reach significance (Figure 2E).

**Figure 3:**
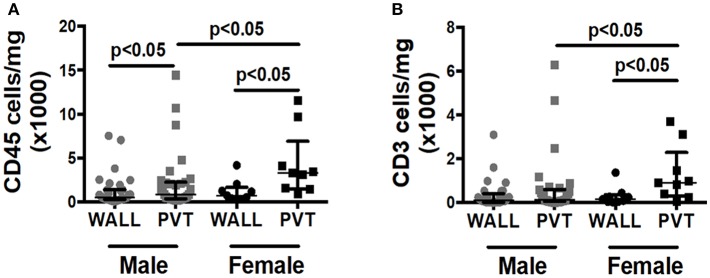
Effect of patient gender on immune cell numbers in AAA wall and PVT. Male vs. female total leukocyte and T cell content in AAA PVT and wall. **(A)** Leukocyte number per mg of aneurysmal wall and PVT in male group *n* = 31 vs. female group *n* = 9 **(B)**. T cell number per mg of aneurysmal wall and PVT in male group *n* = 30 vs. female group *n* = 9. Mann-Whitney test for comparison male vs. female for wall and PVT, Wilcoxon test for comparison wall vs. PVT within male and female group. *p* values presented on graphs only for statistically significant comparisons.

Although over 80% of our patient samples derived from males, we were curious if we could observe any gender differences in total leukocyte or T cell counts within patient samples. Interestingly, female patients displayed significantly more leukocytes and T cells within the PVT in comparison to male patients (Figures 3A,B).

### Activated and Immunosenescent T Cells in AAA

Next, we investigated the activation status of aneurysmal T cells by utilizing the activation markers: CD69, CD25, HLA-DR as well as CCR5 and the absence of CD28 (CD28^null^; representing immunosenescent phenotype). We revealed that CD4^+^ T cells are preferentially activated within the aneurysmal wall (Supplemental Table 2A). More than 50% of T cells expressed the early activation marker CD69 in AAA, either in PVT or in wall; with a higher percentage of CD4^+^CD69^+^ cells in aneurysmal wall. This was in line with higher percentages of CD4^+^T cells expressing the late activation marker HLA-DR in AAA wall than in aneurysmal PVT (Supplemental Table 2A). Interestingly, while CD8 cells within both AAA wall and PVT were highly activated, they did not differ between wall and PVT (Supplemental Table 2B). Similar to markers of activation, there was an increased presence of CD4^+^CCR5^+^ cells in aneurysmal wall compared with PVT, which was not observed in the CD8^+^ T cell population, although notably CCR5 was present on over 40% of all CD8 cells present either in AAA wall or PVT (Supplemental Table 2B).

In contrast to the above, the percentages of immunosenescent, dysregulated CD8^+^CD28^null^ T cells were higher in AAA wall than in PVT whereas CD4^+^CD28^null^ cells did not differ between aneurysmal PVT and wall (Supplemental Tables 2A,B).

### T Cell Infiltration and AAA Size

**Figure 4:**
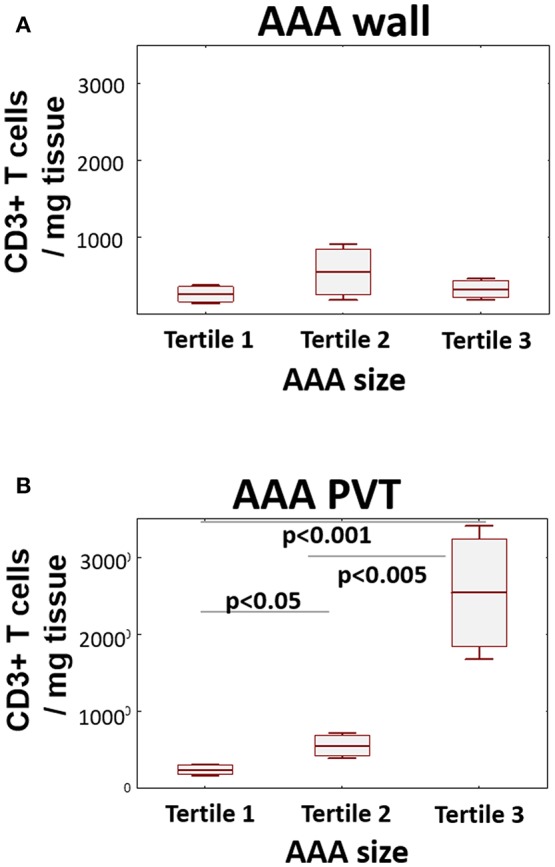
Relationship between T cell number and AAA size. AAA diameter was determined by preoperative CT. Graphs display relationship between AAA size (tertile 1 ≤ 53 mm; tertile 2 ≤ 60 mm; tertile 3 > 60 mm) and CD3^+^ cell infiltration in wall **(A)** and PVT **(B)** (mean/SEM/25–75 CI); (*n* = 20/10/9 for tertiles); statistical comparisons were performed by ANOVA with Neuman-Keuls *post-hoc* analysis.

Finally, to ascertain if T cell infiltration is associated with AAA stage/severity, we stratified T cell content in both wall and PVT according to AAA diameter (Figure 4). While no significant differences in T cell content were observed in AAA wall, significant differences in T cell content were seen in PVT with the highest tertile containing the greatest number of T cells while the lowest tertile had PVT T cell numbers comparable with wall.

## Discussion

In this investigation, we provide novel quantitative data on leukocyte populations in late-stage AAA, which identified T cells as the dominant immune cell population in these vessels. Moreover, we identified that PVT is a key site of T cell accumulation in AAA with T cell numbers increasing with AAA size, with these conclusions based on analysis of a large cohort of AAA samples. This suggests that the pro-inflammatory role of PVT in the development of vascular pathology (40–43) can now be extended to late-stage AAAs.

Our study provides an important quantitative assessment and clinical context to previous immunohistochemical and gene expression reports indicating a potentially important role for the abnormal necrotic, inflamed, proteolytic tissue adjacent to the aneurysmal wall in regulating ongoing vascular damage. Indeed, inflammatory cells such as neutrophils, T cells and others often surround necrotic adipocytes (38). The role of T cells in this process remains unclear. Using detailed, quantitative flow cytometry studies, we have demonstrated that the AAA T cell population expresses characteristic activation markers. While there are significantly more leukocytes and T cells in the PVT, cells in the pathologically damaged AAA wall are more activated (especially CD4^+^ cells) or represent dysregulated, immunosenescent CD28^null^ phenotype (particularly CD8^+^ cells). This is interesting, as we have recently shown that CD8^+^CD28^null^CD57^+^ cells are important in the immediate response to vascular injury (44). These cells are known to produce increased amounts of pro-inflammatory TNF-α and IFN-γ further contributing to vascular inflammation. Therefore, this population may be important in AAA pathology with percentages of peripheral blood CD8^+^CD28^null^ cells being higher in AAA patients than controls but without a clear relationship to the maximal aneurysm diameter (24).

In spite of these differences, we observed a positive correlation between the numbers of both total CD45^+^ leukocytes and in particular T cells between PVT and AAA wall. This may be particularly relevant in the context of the debate regarding the source and trafficking of leukocytes to AAAs. Considering this, abluminal white thrombus which is located directly on the internal surface of the AAA wall, shows negligible numbers of leukocytes (45). The data we present here show an abundance of leukocytes in PVT which may suggest that trafficking of immune cells into the AAA wall may be secondary to their entry into the PVT, possibly via entry through PVAT associated post-capillary venules (38). Further support for PVAT recruitment of T cells came from the discovery that human adipocytes in PVAT surrounding atherosclerotic arteries produce chemoattractants and are able to induce chemotaxis of leukocytes including T cells (46). T cells are also found densely aggregated within the PVT in an angiotensin induced mouse model of hypertension (47) suggesting an “outside-in” infiltration into the vessel. However, in AAA samples, which contain adventitial neoangiogenesis, it may also be the case that T cells are recruited via the adventitial microvasculature, depending on the local cellular and cytokine environment. We used both flow cytometry and IHC to compare content and localization, respectively, for both macrophages and T cells in PVT and wall. T cells comprised a significantly greater proportion of leukocytes in both compartments with T cell numbers being highest of all in PVT. IHC analysis also revealed dense T cell staining within the PVT. This is somewhat expected considering this is the location where lymphocyte aggregates develop in AAA patients (48).

Previously, it was suggested that CD4^+^ T cells may play a crucial role in AAA formation in both animal models of disease and human pathology (12, 23, 30). Our study supports this view, by demonstrating that CD4^+^ T cells are the major T cell subtype in AAA. Despite leukocytes, including T cells, being constitutively present in healthy aorta (49), infiltration of Th1 CD4^+^ T cells from the adventitia into the media can distinguish thoracic aneurysmal aorta segments from non-aneurysmal dilated aorta (25). Our data now suggest that infiltrating cells are preferentially recruited to the PVT, where they are densely dispersed throughout the tissue layer, often in close proximity to less abundant PVT macrophages. While the results of such interactions are unclear in our samples, activated T cells have been shown to promote the release of macrophage derived pro-inflammatory factors in mouse models of AAA (30). Activated CD4^+^ Th1 cells are the dominant T cell in atherosclerotic murine aorta and have previously been shown to predominate in human AAA samples (23). Our data now indicates that PVT is a major reservoir of these cells. We also expand on these earlier findings by demonstrating that the majority of CD4^+^ and CD8^+^ T cells present within the aneurysm positively express CD69 as well as showing expression of other activation markers including HLA-DR^+^ and CCR5.

Elevated expression of CCR5 in PVT containing CD4^+^ and CD8^+^ cells as well as a differential expression of these cells between AAA wall and PVT (seen for CD4^+^ T cells) provides a hint for the role of RANTES chemokine in T cell recruitment to PVT and AAA wall. This data, coupled with the fact that RANTES is upregulated within the adventitia of human AAA (8) may suggest a role for this chemokine in the regulation of T cell trafficking in AAA, which has been demonstrated in relation to many other risk factors of AAA such as hypertension (50). Furthermore, angiotensin II induced expression of RANTES within the vasculature and CCR5 on T cells is thought to mediate T cell accumulation within the PVAT and adventitia in a mouse model of hypertension (47) indicating the importance of this chemotactic axis in both mice and humans.

While the focus of our investigation was not T regulatory cells (Tregs), the relatively low frequency of CD25 expressed by both CD4^+^ and CD8^+^ T cells suggest Tregs were not abundant within either AAA wall or PVT. A protective role for Tregs has been demonstrated in animal models of AAA (51, 52) and loss of Tregs correlate with AAA severity (53). It can be envisaged that a shift in T cell subset balance occurs during the chronic inflammation associated with AAA resulting in a loss of T regulatory function with concomitant enhancement of pro-inflammatory CD4^+^ Th1 and Th17 cells (54).

We established a relationship between T cell number and AAA size for PVT but not for AAA wall indicating that PVT recruits (and/or retains) more T cells as AAA progresses in severity. The fact that T cells are more activated within the wall may suggest greater retention of highly activated cells within the wall compared with PVT or additional inflammatory cues are present that promotes activation of T cells locally. While the precise triggers and role for T cell mediated immune responses in human AAA remain unclear, the presence of T and B cell aggregates within AAA (or more complex artery tertiary lymphoid organs; ATLOs) are suggestive of an autoreactive immune response to unidentified self/modified self-antigens (18, 55). Local clonal expansion of T cells within AAA remains a matter of debate. While the function of ATLOs in human AAA remains unclear, they are closely linked to adaptive immune activation and may feasibly occur as a progression of earlier aberrant autoreactive immune responses resulting in a highly compartmentalized local immune response (56).

It is important to emphasize that we have studied advanced AAA disease. With increasing use of endovascular aortic repair only more advanced cases are undergoing an open operative repair. This may be a source of selection bias we should take into account when interpreting these results.

In summary, we demonstrate that the majority of immune cells in late-stage human AAA are present within the PVTs with a predominating presence of T cells. Activation of T cells is apparent within PVT with activation status increasing still further in AAA wall populations. We also provide evidence that T cell content in PVT is enhanced with increasing maximal AAA dilatation, highlighting a potential important role for PVT inflammation in AAA pathology.

## Data Availability

The datasets generated for this study are available on request to the corresponding author.

## Ethics Statement

Written informed consent was obtained from all patients. Collection of tissue was approved by the local Research Ethics Committee of the Jagiellonian University, Kraków, Poland (Approval No. KBET/78/B/2012) and the West of Scotland Research Ethics Service Committee for the Biorepository at the Queen Elizabeth Hospital, Glasgow, United Kingdom (Approval No. 10/S/0704/60).

## Author Contributions

AS and TM designed and performed the experiments and wrote the manuscript. WM, KD, and AM designed clinical part of the study and obtained the clinical samples. CD participated in the sample collection. NM analyzed the data and wrote the manuscript. SM performed flow cytometric analysis and analyzed immunofluorescence. KU developed the methodology, performed the experiments, and analyzed the data. KD, AM, GF, and BK performed the clinical part of experiments. PM interpreted the data and wrote the manuscript. RT designed the experiments and provided comments on the manuscript. TG designed the experiments, obtained the clinical data, and wrote the manuscript. All authors have read and approved the final manuscript.

### Conflict of Interest Statement

The authors declare that the research was conducted in the absence of any commercial or financial relationships that could be construed as a potential conflict of interest.
