# Supplementary material for: T Cells Are Dominant Population in Human Abdominal Aortic Aneurysms and Their Infiltration in the Perivascular Tissue Correlates With Disease Severity
Source: Front Immunol. 2019 Sep 4;10:1979. doi: 10.3389/fimmu.2019.01979 (PMC6736986; doi:10.3389/fimmu.2019.01979)
Supplement: Supplementary file 1 [file Data_Sheet_1.docx]

**Supplementary Data**

**T Cells Are Dominant Population in Human Abdominal Aortic Aneurysms and Their Infiltration in the Perivascular Tissue Correlates with Disease Severity**

Agnieszka Sagan^1,2^, Tomasz P. Mikolajczyk^2,3^, Wojciech Mrowiecki^4^, Neil MacRitchie^3^, Kevin Daly^5^, Alan Meldrum^5^, Serena Migliarino^1^, Christian Delles^1^, Karol Urbanski^2^, Grzegorz Filip^6^, Boguslaw Kapelak^6,7^, Pasquale Maffia^3,1,8^, Rhian Touyz^1^, Tomasz J. Guzik^1,2,*^

**Supplemental Figure 1. Cell numbers in AAA wall and PVT.**  Absolute cell number per mg of aneurysmal tissue wall vs PVT, n=40, Wilcoxon matched paired.

**Supplemental Table 1.** Antibody clones employed for flow cytometry analysis.

| **Antibody** | **Clone** | **Manufacturer** |
| --- | --- | --- |
| CD45-PeCy7 | HI30 | BD Biosciences |
| CD3-PerCP | SK7 | BD Biosciences |
| CD4-PeCy7 | SK3 | BD Biosciences |
| CD8-APC-H7 | SK1 | BD Biosciences |
| CD69-FITC | FN50 | BD Biosciences |
| CD25-PE | M-A251 | BD Biosciences |
| HLA-DR-Pe-Cy7 | L243 | BD Biosciences |
| CD195-PE | 2D7/CCR5 | BD Biosciences |
| CD28-APC | CD28.2 | BD Biosciences |
| CD45-APC | HI30 | BioLegend |
| CD3-AF488 | UCHT1 | BioLegend |
| CD19-Pe-Cy7 | HIB19 | BioLegend |
| CD64-PE | 10.1 | BioLegend |
| CD11b-APC-Cy7 | ICRF44 | BioLegend |
| CD66b-PE | G10F5 | BioLegend |
| CD83-APC-Cy7 | HB15e | BioLegend |
| CD16-Pe-Cy7 | 3G8 | BioLegend |
| CD56-AF488 | HCD56 | BioLegend |

**Supplemental Table 2A.** Expression of CD69, CD25, HLA-DR, CCR5 and CD28 antigens in CD4+ T cell in the abdominal aortic aneurismal wall and PVT.

| **%**  **(MFI)** | | **WALL AAA** | **PVT AAA** | **p** | **N** |
| --- | --- | --- | --- | --- | --- |
| **CD4+** | **CD69+** | **58.4±2.4**  (1332±161) | **50.3±2.5**  (1178±112) | **0.01**  NS | **18**  18 |
|  | CD25+ | 10.3±2  (431±27) | 12.9±2.2  (438±58) | NS  NS | 15  14 |
|  | **HLA-DR+** | **31.6±4.7**  (1748±219) | **16.2±1.1**  (1748±296) | **0.01**  NS | **22**  22 |
|  | **CCR5+** | **25.4±3.2**  (987±114) | **16.7±2.6**  (1030±102) | **0.02**  NS | **22**  22 |
|  | CD28null | 12.5±3.4 | 12±2 | NS | 15 |

**Supplemental Table 2B.** Expression of CD69, CD25, HLA-DR, CCR5 and CD28 antigens in CD8+ T cell in the abdominal aortic aneurismal wall and PVT.

| **%**  **(MFI)** | | **WALL AAA** | **PVT AAA** | **p** | **N** |
| --- | --- | --- | --- | --- | --- |
| **CD8+** | CD69+ | 55.9±2.5  **(1326±200)** | 53.7±2.9  **(1106±134)** | NS  **0.03** | 18  **18** |
|  | CD25+ | 7.9±1.7  (511±67) | 9.2±1.6  (500±64) | NS  NS | 15  15 |
|  | HLA-DR+ | 28±2.9  (1428±105) | 27.7±2.1  (1427±85) | NS  NS | 22  22 |
|  | CCR5+ | 48.9±4  (1100**±**154) | 43.8±4.2  (1132**±**140) | NS  NS | 22  22 |
|  | **CD28null** | **30.5±7.9** | **19.5±4.3** | **0.01** | **15** |
